# Automatic Segmentation of Dermoscopic Images by Iterative Classification

**DOI:** 10.1155/2011/972648

**Published:** 2011-07-17

**Authors:** Maciel Zortea, Stein Olav Skrøvseth, Thomas R. Schopf, Herbert M. Kirchesch, Fred Godtliebsen

**Affiliations:** ^1^Department of Mathematics and Statistics, University of Tromsø, 9037 Tromsø, Norway; ^2^Norwegian Centre for Integrated Care and Telemedicine, University Hospital of North Norway, 9038 Tromsø, Norway; ^3^Venloer Straße 107, 50259 Pulheim, Germany

## Abstract

Accurate detection of the borders of skin lesions is a vital first step for computer aided diagnostic systems. This paper presents a novel automatic approach to segmentation of skin lesions that is particularly suitable for analysis of dermoscopic images. Assumptions about the image acquisition, in particular, the approximate location and color, are used to derive an automatic rule to select small seed regions, likely to correspond to samples of skin and the lesion of interest. The seed regions are used as initial training samples, and the lesion segmentation problem is treated as binary classification problem. An iterative hybrid classification strategy, based on a weighted combination of estimated posteriors of a linear and quadratic classifier, is used to update both the automatically selected training samples and the segmentation, increasing reliability and final accuracy, especially for those challenging images, where the contrast between the background skin and lesion is low.

## 1. Introduction

Melanoma is a common cancer in the adult population, and accounts for a considerable number of deaths in fair skinned people worldwide. It may arise within preexistent moles or *de novo* in unaffected skin. When diagnosed at an early-stage prognosis is excellent, the melanoma can be cured by simple excision. However, as melanomas can be hard to distinguish from common moles, even for experienced dermatologists, early diagnosis is a challenge, especially for general practitioners. 

A recent advance in diagnosis of melanomas is the emergence of dermoscopy, also known as epiluminescence microscopy. Dermoscopy is a noninvasive diagnostic technique that consists in the examination of skin lesions with a dermoscope, which is a hand held optical device that typically consists of a magnifying lens and a light source, used to illuminate the skin. Usually, a transparent plate and liquid medium is placed between the instrument and the skin, allowing the inspection of a lesion, in the upper layers of the skin, in great detail. This clear view of the skin can help with diagnosing skin cancers [1].

There is a considerable variation in the appearance of common moles and melanomas. They both appear as spots on the skin, with diameters ranging from a few millimeters up to several centimeters. Color typically is brown or black, but red and blue also exists. In most situations the pigmented lesion can easily be identified from the normal surrounding skin which appears lighter.

Delineation of the contour of pigmented skin lesions (segmentation) plays a relevant role for automatic feature extraction, where the purpose is automatic diagnosis of melanomas. Image segmentation is the process of adequately grouping pixels into a few regions, whose pixels share some similar characteristic, like color, texture, or shape [2]. Automated analysis of edges, colors, and shape of the lesion relies upon an accurate segmentation and is an important first step in any computer aided diagnosis system. Indeed, most commercially available systems show a great variability in reliability and specificity in the diagnostic process, and the image segmentation also varies greatly [3]. Irregular shape, nonuniform color, and ambiguous structures make the problem challenging [4]. The presence of hairs and skin flakes are additional undesirable features that may interfere with segmentation. An additional complication arises in the validation of any technique, as there is no gold standard to refer to. Even trained dermatologists differ significantly when delineating the same lesion in separate incidents [5], so validation of any technique has to be treated with care. Indeed, an important feature of any segmentation procedure must be reproducibility. Even under the best effort to counter this, different images of the same lesion will differ slightly in illumination, rotation, and shear due to the flexibility of the skin.

Supervised segmentation methods are those methods that require input from the analyst, such as examples of skin and lesion pixels, a rough approximation of the lesion borders to be optimized, or a final refinement of a proposed solution [6–8]. Generally, in such settings, the user needs to provide a priori input for each particular image being analyzed. This task relies on the experience and knowledge of the user. Although this kind of approach may be very effective, the process may be particularly time consuming for health care professionals. As an alternative, automatic segmentation methods (also called unsupervised methods) attempts to find the lesion borders without any input from the analyst and can, therefore, be applied even by persons who are not trained in dermatology. Several approaches have been proposed in this direction. Most common automatic segmentation algorithms rely on techniques based on histogram thresholding [7, 9–12], where most commonly RGB information is mapped to a one or two-dimensional color space through choice of one of the channels, luminance, or principal component analysis. Other approaches include clustering [4, 13, 14], region-based techniques [7, 15, 16], contour-based approaches [7, 17–20], genetic programming [21], and segmentation fusion techniques [22]. A recent overview of methods applied to segmentation of skin lesions in dermoscopy images suggest that clustering is the most popular segmentation method, probably due to the availability of robust algorithms [6].

The algorithm proposed in Celebi et al. (2008) [16] uses a region growing and merging technique called statistical region merging (SRM) [23] to segment the image. SRM was proven to be a robust segmentation algorithm for segmentation of color images. In [16], SRM achieved better results on segmentation of skin lesions when compared to five automated methods: the dermatologist-like tumor extraction algorithm [15], the JSEG segmentation algorithm [24], the mean-shift clustering [4], and the orientation-sensitive fuzzy c-means [13]. The recently proposed automatic adaptive thresholding (AT) by Silveira et al. (2009) [7] segments the image by comparing the color of each pixel with a threshold. The algorithm uses entropy to find the most suitable RGB channel for discrimination, and a pixel is classified as lesion if it is darker than the threshold. Distinct rules are proposed to compute the threshold for the cases of bimodal or single component histograms.

Currently, several instruments designed for computer aided diagnosis (CAD) of skin lesions are commercially available. Despite the use of powerful and dedicated video cameras, the cost related to the acquisition material [25] or the actual usefulness of these instruments for dermatologists practising [3] may be the reasons that prevent their wide diffusion to physicians.

At the other hand, current limitations of state-of-art CAD instruments motivates the development of new algorithms for analysis of skin lesions and simple data acquisition options. Following an approach that might be practical and intuitive for dermatologists, the images considered in this study are taken by a consumer digital camera with a dermatoscope attached. This simple image acquisition setup was considered for instance in [26–28]. As a usual procedure for dermatologists, an alcohol-based contact fluid liquid between the skin and the dermoscope is used during the acquisition of the images. This minimizes the formation of air bubbles, reducing artifacts.

Although several algorithms for unsupervised image segmentation have been proposed in the literature, they do not necessarily perform well for the specific problem of skin lesion segmentation, and it appears unlikely that one particular method could outperform all other segmentation methods for any lesion. Our idea is to exploit (a) the fact that skin lesions are approximately located in the center of the dermoscope during the acquisition process and (b) the color characteristics of typical lesions and integrate such information into a reliable framework for automated segmentation of skin lesions. This results in an algorithm for segmentation based on classification, where the initial training data set is selected as portions of the image based on these initial assumptions, and the training set is iteratively expanded. This training and classification occurs within one image only, the training set being small portions of the image called seed regions. Thus, we arrive at our proposed algorithm, which we will denote iterative classification Segmentation (ICS).

The proposed segmentation method introduces novelties in the current state-of-art of methods designed specifically for segmentation of dermoscopic skin lesions.

1. An automated method is proposed to select small seed regions to represent both the background skin and lesion. In the proposed approach, seed regions are used for initialization of an iterative classification framework. This differs from previous studies, in some of which seed regions are manually placed for modeling the background skin [4], or others, where it is assumed that the corners of the lesion images are good estimates for the background skin [16]. We believe our approach provides more flexibility in the search for suitable regions despite its simplicity.
2. The lesion segmentation problem is treated as a binary classification problem. A hybrid strategy that combines classification posterior probability estimates from two distinct classifiers is presented. For each particular lesion, the best choice between a linear or quadratic classifier (or a weighted combination of both) is automatically set by optimization of the classification accuracy. The proposed classifier proved to be valuable in the context of iterative classification.
3. An iterative approach is used to update the parameters of the classifiers and the final segmentation. In contrast to semisupervised learning techniques that make use of both labeled and unlabeled data for training [29], where typically high confident samples are iteratively added to the initial set of ground truth manually labeled samples; here, the proposed method attempts to use only automatically selected samples with no supervision involved. It appears of interest to understand if such kind of approach would be valuable to solve the problem of segmentation of skin lesions.
4. The proposed method makes specific hypothesis for the segmentation problem of interest, allowing the design of safeguard criteria for driving the unsupervised update of training samples. We believe that the idea might also be successfully extended to segmentation of other kind of medical images, provided that the hypothesis, notably the a priori location of and relative color of elements of interests, is properly defined.
5. As with most of methods dealing with image processing, there are inevitably parameters that must be set by the user. Throughout this paper, we attempt to use parameters defined in length units as much as possible, thus using the magnitude of a physical quantity. Since in practical applications, the resolution of the equipment used for image acquisition can be measured, the parameters can be easily converted to pixel unities for image analysis. Surprisingly, we observe in the literature that most papers dealing with segmentation of skin lesions cite the relevant algorithm parameters in pixels, or a value relative to the image size, often without reference to the image resolution. This complicates unbiased implementation of alternative techniques for comparison purposes. Hopefully, our approach might help other researchers using different equipment.

The remaining of the paper is organized as follows; in Section 2, we introduce the ICS framework.  Section 3 outlines the postprocessing steps allowing the final segmentation.  Section 4 presents experimental evidence of the competitiveness of the ICS algorithm for segmentation of skin lesions using dermoscopy images. We conclude with final remarks in Section 5.

## 2. Methodology

The proposed ICS framework is characterized by three main processing stages.

1. Initialization: automatic search for seed regions under assumptions on the approximate location of the lesion and the usual lighter nature of the skin color compared to the lesion. The seed regions provide training samples for the binary classification between background skin and lesion.
2. Classification: two distinct base classifiers are made available to the classification procedure, specifically a linear and quadratic classifier. The decision of which classifier to use for each particular lesion, or possibly a combination of both classifiers (weighting), is automatically decided using optimization of the classification accuracy. This classification strategy can be seen as a hybrid classifier, here defined in terms of the combination of the posterior classification probabilities. The classification is iterated, facilitating robust selection of new training samples and segmentation.
3. Iteration: the automatically selected training samples are updated and the classification is repeated iteratively until convergence.

**Figure 1:**
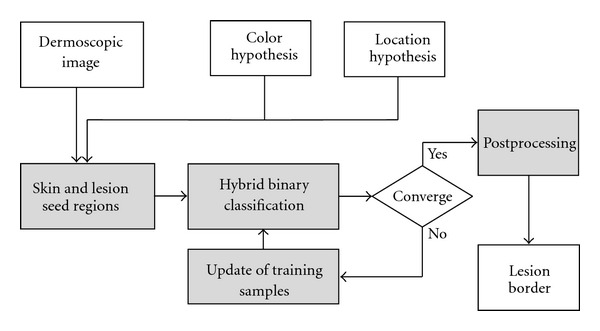
A flowchart of the proposed ICS algorithm showing the main processing modules.

A flowchart of the proposed ICS algorithm is depicted in Figure 1. A detailed description of the assumptions and the relevant processing stages is provided in Sections 2.2–2.5. The postprocessing that leads to the final segmentation mask is presented in Section 3. 

### 2.1. Preprocessing

The RGB image is first processed to the perceptual uniformity CIE *L*∗*u*∗*v* color space. The *L*∗*u*∗*v* color space attempts perceptual uniformity, and it is extensively used for applications such as computer graphics and was previously used for segmentation of skin lesions [13].

### 2.2. Assumptions

Specific assumptions about the dermoscopic image acquisition and the colors of skin and lesion are used for the initial automatic selection of seed regions, as described below.

#### 2.2.1. Image Acquisition

**Figure 2:**
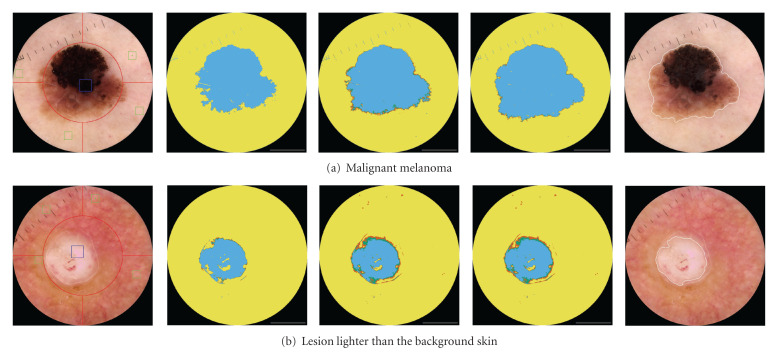
(Left) The small automatically selected green boxes correspond to the skin seed regions used as initial training. The location is based on the brightest luminance in each quadrant. The blue box corresponds to the seed region for the lesion, selected as the region statistically most different from the skin seeds. The three middle images show intermediate iterative steps at iterations *j* = {1,2} and the final iteration which is *j* = 7 in (a), *j* = 3 in (b). In these maps, yellow and blue pixels are those classified with high confidence as skin and lesion, respectively. The maps include also red and green pixels, corresponding to those pixels classified with low confidence as skin and lesion, respectively. The white contour in the rightmost figure is the final border obtained after postprocessing.

We assume that at least part of the lesion is located inside the circular spatial domain *d*_1_ = {*r* ≤ 5 mm}, where *r* is the radius of a disk centered in the image (*r* = 5 mm corresponds to the red circular ring in Figure 2). In practice, this assumes that some care was taken during the acquisition of the image. The assumption is not very stringent: as it will be described in Section 2.3, only a small part of the lesion needs to be located inside this spatial domain. When the lesion is relatively small, the domain *d*_1_ might enclose the entire lesion.

In a similar way, we assume that in the remaining part of the imaged area, the spatial domain *d*_2_ = {5 mm < *r* ≤ *ℓ*}, where *ℓ* is the radius of the entire circular imaged area acquired using the dermatoscope (with our equipment, *ℓ* ≈ 8.7 mm), a few small skin regions, possibly spatially homogeneous, are available. In general, this should be true, except for big lesions covering the entire image. For skin lesions covering the full dermoscopic area, (i.e., *≈*240 mm^2^ with the current dermatoscope), segmentation between skin and mole is not applicable. From a medical point of view, the size of a lesion is itself indicative of further medical attention, but since segmentation is meaningless in such cases, we will ignore these in the remainder.

#### 2.2.2. Skin Color

An a priori assumption regarding the color of both skin and lesion areas is necessary to initialize the unsupervised (automatic) selection of training samples. We assume that the lighter areas in *d*_2_ are likely to be skin and those areas in *d*_1_ that differs most, in a statistical sense, from the skin correspond to lesion.

**Figure 3:**
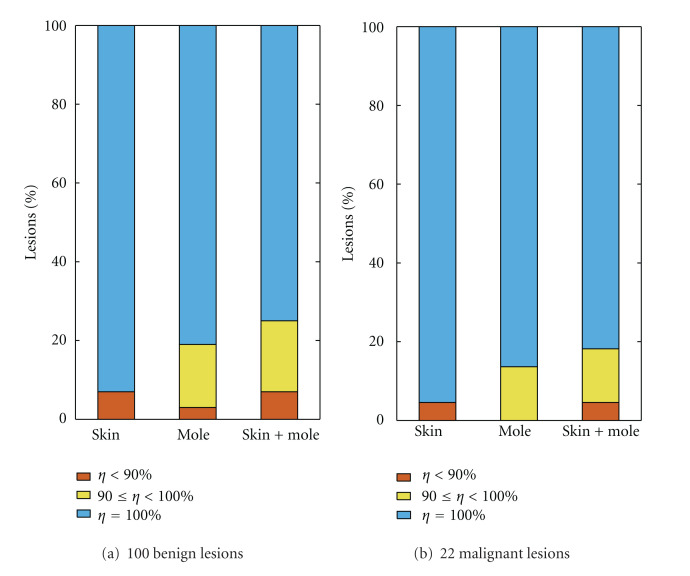
Average accuracy (*η*) of the detected seed regions for the images in the test set, according to the diagnosis. For visualization purposes, instead of the number of lesions, the vertical axis shows the corresponding percentage out of 100 benign and 22 malignant lesions.

The statistical difference is here computed measuring the Euclidian distances in the Luminance component of the calibrated RGB image processed to the CIE *L*∗*u*∗*v* color space. As will be shown later in experiments (in Figure 3), the location of the seed regions using only the Luminance component proved to be robust enough to the great majority of lesions analyzed. 

### 2.3. Stage1: Initial Unsupervised Selection of Training Samples

#### 2.3.1. Skin Samples

The peripheral spatial domain *d*_2_ is divided into four quadrants. In each quadrant, we search for a small seed, a box of size *w*_skin_ × *w*_skin_ pixels corresponding to the lighter colored areas by looking for the regions with maximum average value of the pixel's luminance component. According to the assumption in Section 2.2.2, these regions are likely to be skin portions. We suggest *w*_skin_ = 1.0 mm. The skin seed regions corresponds to the green boxes depicted in green in Figure 2.

Once the four skin seeds are positioned, in order to further increase the robustness of the process, we select and merge only the three seeds that correspond to the regions that are statistically most similar by testing all three vs one combination of regions (again, measuring the Euclidian distance of the luminance component in the two sets). The pixels located in the seed regions are the initial skin training samples. Excluding one out of the four seeds reduces the risk of incorporating regions that are contaminated by artifacts.

#### 2.3.2. Lesion Samples

Once representative initial skin samples are identified (Section 2.3.1), we search in the central spatial domain *d*_1_ for the box of size *w*_mole_ × *w*_mole_ whose average pixel values in the luminance component has the largest Euclidian distance from the initial skin training samples average value.

Depending on the size of the box compared to the size of the lesion, skin pixels may be present inside the box. Intuitively, a bigger box over the lesion would better capture the variability of the lesion, but it increases the probability of including skin pixels, so a tradeoff must be made. We suggest *w*_mole_ = 1.5 mm. Examples of this initial location for the lesion seed are the blue boxes shown in Figure 2.

For illustrative purposes, Figure 2(b) includes the case of a challenging lesion characterized by a brighter color compared to the background skin. In this particular example, the proposed method was able to accurately position both seed regions. 

### 2.4. Stage 2: Classification

Once the initial training samples are automatically selected using the procedure described in the previous section, the samples can be used for “supervised” classification. Note that the initial training samples were obtained in an automated way using only assumptions about the images. Notice, however, that since the initial training samples were obtained from an automatic strategy, a mechanism aiming at improving the segmentation accuracy and the confidence of the automatic training samples selection would be desirable. 

From a classification perspective, it is worth mentioning that the initial seed regions (the automatically selected skin and mole box regions in Figure 2 using the luminance *L* component) are likely to be perfectly separable. This is because the seeds are found by looking for bright pixels on the periphery and the most dissimilar pixels in the center. Therefore, it is likely that the decision boundary will be poorly specified and hence unlikely that a quadratic classifier could initially outperform a simple linear classifier. However, as new training samples are iteratively added to the model, and using all the *L*∗*u*∗*v* color components, one could expect that the decision boundaries between both skin and lesion would become progressively better defined in the feature space and potentially allowing a quadratic classifier to benefit from the higher flexibility in placing the discriminating boundaries.

Because of their simplicity and fast calculation, linear discriminant analysis (LDA) and quadratic discriminant analysis (QDA) [30] are initially considered as base classifiers for the binary classification problem. QDA fits multivariate normal densities with covariance estimates stratified by lesion and skin groups, when LDA uses a pooled covariance matrix estimation. We also considered using a nonparametric classifier (specifically, the neatest-neighbor classifier), but due to the computational burden it was discarded. LDA can be seen as a particular case of the QDA classifier, raising the question if there is some added value in considering the use of a linear classifier in the proposed method. In preliminary experiments, we found scenarios where the simple linear choice appears to be preferable, for instance, in cases where the seed regions corresponding to skin are placed on very homogenous areas. This areas typically exhibit very low variance, compromising the estimation of the covariance matrix for the skin class in QDA. The low variance may result also from the placement of the seed in regions that does not provide an accurate representation of the statistics of all the background skin (poor location). If the quadratic classifier is used, despite the perceptual small differences of color between the seed region for skin and the whole background skin, it appeared that skin portions would be wrongly classified as high confident skin (*p*_*k*_^*λ*^(*y* = 1 | *x*) > 0.98). This undesired behavior would compromise the iterative update of confident training samples, especially for the lesion. Especially at the beginning of the iterative classification, it appears to be more prudent to use of linear classifier. As more samples are added, a better estimate of the class statistics would be a benefit for the quadratic classifier.

The above considerations suggests that the use of an iterative procedure would be a reasonable choice for update of the initially selected training samples, and ideally, the choice between LDA and QDA should be automatic in the iterative process.

Our proposed methodology for selection between LDA and QDA for classification is very simple in nature. Assume that the classification output for a given pixel *x* is *y* = 1 for pixels classified as skin and *y* = 2 for lesion. We consider a class of hybrid posterior estimates

(1) $$\begin{array}{cc} p_{k}\left(y=\Omega \;|\;x\right) & =\lambda_{k}p_{k}^{\text{QDA}}\left(y=\Omega \;|\;x\right)\\ & \;+\left(1-\lambda_{k}\right)p_{k}^{\text{LDA}}\left(y=\Omega \;|\;x\right), \end{array}$$

where 0 ≤ *λ*_*k*_ ≤ 1. Given the training data, our aim is to choose a value of *λ*_*k*_ that maximizes classification accuracy of classes *Ω* = {1,2} at each iteration *k* = {1,2, 3,…, *n*}.

Without ground-truth samples, estimating classification accuracy is a challenging task. We rely on the training samples from the automatically placed seed regions. Such samples are dived in two spatially disjoint subsets. One subset is used to train LDA and the other QDA. In practice, one and a half of the three seed regions corresponding to skin, and half of the mole seed region are used in each classifier. This reduces the spatial correlation between the samples used in both classifiers. For an easy implementation of (1), the interval [0 − 1] for *λ*_*k*_ is finely partitioned and the value of *λ*_*k*_ that provides the best classification is chosen. For each value of *λ*_*k*_, we compute the classification accuracy for both the skin and the lesion classes and retain the minimum value. At least, half of the samples remained unseen by each classifier, acting like independent test samples. An important point is that in case of identical classification accuracies for distinct values of *λ*_*k*_, the lowest value of *λ*_*k*_ is selected, privileging the simpler LDA. Typically, this is the case in the first iterations. 

### 2.5. Stage 3: Iterative Update of Training Samples and Classification

After the initial binary classification, new additional samples are added iteratively to the classification process to better estimate the parameters of the classification model. In order to keep classification stable, it is desirable to focus on those skin and mole pixels classified with high confidence: those pixels classified with an estimated a posterior probability higher than the predefined threshold *p*_*k*_^*λ*^(*y* = *Ω* | *x*) > *τ*. In our experiments, we set *τ* = 0.98, which seems a reasonable empirical choice. In the case of the background skin, any position in the image classified as skin with high confidence is eligible for providing additional training samples. In the case of the lesion, an additional safeguard constraint is imposed. The idea is to eliminate artifacts and lesion pixel candidates from small isolated regions that are possibly not representative. For this purpose, mathematical morphological opening [31] is applied to regions classified as mole in the input binary mask. In our experiments, opening is applied using a disk of diameter *d* = 1 mm as structural element. In the sequence, the continuous subregion resulting from opening that best encloses the initial lesion seed box is selected. Lesion pixels classified with high confidence inside this subregion are eligible for update. This restriction is not applied for the segmentation itself but only for the selection of training samples.

The classifiers are, thus, iteratively trained using those samples from the original seed regions plus an additional set of training samples randomly selected at each iteration. For practical computational, the maximum number of added samples for each class is limited to the corresponding number of samples contained in the original seed regions. Thus, at each iteration, half of samples are the original, and half are updated with high confident ones. 

The classification and training sample selection is repeated until convergence. The default stop criteria of the iterative update of training samples is defined as

(2) $$\begin{array}{c} \left|1-\frac{m_{k+1}}{m_{k}}\right|<0.01 \end{array},$$

where *m*_*k*_ is the number of pixels classified as lesion with high confidence at iteration *k*.

## 3. Postprocessing

The final binary segmentation mask might contain a certain number of disjoint regions classified as lesion. Ideally, the segmentation procedure is expected to produce two independent regions: lesion and background. Since these regions are rarely homogeneous, segmentation can classify multiple isolated objects as lesion. To obtain a single lesion object, a set of postprocessing steps are applied.

First, mathematical morphological erosion [31] is applied to the binary mask obtained from classification. Erosion is commonly used in image processing to eliminate small isolated regions and artifacts. In our experiments, erosion is applied using a disk of diameter *d* = 0.12 mm as structural element. The size of this element seems to be a safe choice [32] when the intention is to eliminate potential isolated hairs classified as mole pixels over the background skin. Morphological dilation [31] is applied to the resulting “cleaned” image. In order to better preserve the fine details of the border, otherwise eliminated by the morphological operations, a slightly bigger disk element of size *d* = 0.3 mm is used for the dilation, and the resulting image is multiplied pixelwise by the input binary mask obtained from classification.

In sequence, the remaining objects in the generated binary mask that are spatially closer than a given threshold are connected. The idea is that small scattered objects classified as mole could be part of a continuous subregion, that eventually could be connected to a big segment, if close together. Connecting regions classified as mole might also be useful for those patients with light-colored hairs imaged over the lesion, likely to be classified as background skin, thus “breaking” the main lesion into subregions during the classification. Again, this can be easily done using a classical spatial image filtering procedure. A Gaussian filter is selected for our experiments. The bandwidth is set identical to the radius used in the above erosion procedure, and the empirical selected value 0.1 is used as threshold for generating the final binary mask containing a certain number of subregions. 

Each contiguous subregion *i* labeled as lesion is given a score *n*_*i*_. If each region *i* is labeled *ℛ*_*i*_ each region's score is given by

(3) $$\begin{array}{c} n_{i}=\underset{u,v\in {ℛ}_{i}}{\sum }f\left(u,v\right), \end{array}$$

where *f*(*u*, *v*) is the two-dimensional Gaussian function

(4) $$\begin{array}{c} f\left(u,v\right)=\exp\;\left\{-\left[\frac{{\left(u-u_{o}\right)}^{2}}{2\sigma_{u}^{2}}+\frac{{\left(v-v_{o}\right)}^{2}}{2\sigma_{v}^{2}}\right]\right\}, \end{array}$$

where (*u*_*o*_, *v*_*o*_) are the coordinates of the center of the disk image, and *σ* is the Gaussian bandwidth. This gives each region a score that increases with size and proximity to the centre, and penalizes regions that are small or off centre. In our experiments, we set *σ*_*u*_ = *σ*_*v*_ = 2.5 mm. This particular choice of *σ* gives over 95% of the total weight to regions (at least partially) centered in a disk of diameter 10 mm. In practice this postprocessing step allows to eliminate potentially disconnected peripheral objects classified as lesion and serves as a guide allowing the doctor to select the lesion to be analyzed by framing the lesion at the center of the picture. 

Except for the region with largest score *n*_*i*_, all remaining objects labeled as lesion are discarded. Any holes in this region are filled. It is worth mentioning that although “single” lesions are more common, there exists also multi-focal lesions. For such particular cases, the current default preferred behaviour of our algorithm is to outline the larger individual lesion. 

Finally, the border of the lesion is drawn around the subregion with the highest score. The Gaussian filter used in the postprocessing renders the contour visually smooth, as usually is the output of hand-drawn borders by dermatologists. See the examples in Figure 2. However, when the border line is intended to be used for computing features for diagnosis in a computer aided diagnosis system, smoothing should be used carefully, since it may be removing information about the contour irregularity, which is an important feature, for example, for the ABCD Rule of Dermatoscopy [33]. 

## 4. Experiments

### 4.1. Data Acquisition

The data used in this study are dermoscopic images acquired by a portable dermoscope (Dermlite Pro II HR) attached to a digital camera (Ricoh GR, Ricoh Company Ltd, Tokyo, Japan). The equipment acquires a circular imaged area of diameter about 17.4 mm (1650 pixels), spatial resolution of about 2400 dpi and 8 bits per channel color depth. The images have been corrected for nonuniform illumination using calibration color standards. A 5 × 5 median filter was applied for noise reduction, but no further preprocessing was used for removing additional artifacts. As mentioned in the introductory section, the images considered in this study were acquired using an alcohol-based contact fluid liquid between the skin and the dermoscope, which minimizes formation of air bubbles in the images. If bubbles were present, it might call for an ad hoc preprocessing algorithm to remove light-colored areas due to reflection in the image that might compromise the skin modeling and location of the seed lesions, with loss of accuracy [6].

 A set of 122 images of pigmented lesions divided between 100 benign and 22 malignant lesions (i.e., melanomas) were used for clinical evaluation of the segmentation. These images were randomly selected from a larger database of dermoscopic images and not used during development of the ICS framework. Copies of the images were printed in 178 × 178 mm paper format and independently given to three dermatologists, who were asked to manually draw the contours of the skin lesions. No additional information, like the histopathological diagnosis, was given to the dermatologists. After careful digitalization, the contour obtained from dermatologists was stored for reference purposes.

It is worth noticing that delineation of the borders of lesions is challenging for dermatologists and is not part of the daily routine, and results can greatly vary between doctors. Essentially, doctors are trained to differentiate between benign and malignant lesions, not necessarily in the specific task of border location.

### 4.2. Measures for Border Detection Evaluation

Qualitative and quantitative approaches are the most common strategies used in literature for the purpose of evaluation of the performance of border detection in dermoscopic images.

Qualitative evaluation of lesion segmentation is a passive strategy in the sense that a candidate border is shown to a dermatologist, who is asked to provide a score or grade to the solution (e.g., good, acceptable, poor, and bad) based on visual assessment. Examples include [19, 34].

In quantitative evaluation, the role of the dermatologist is reversed. In this context, the dermatologist is asked to manually draw the border around the lesion, and the manually drawn border is used as ground truth. Assessing accuracy of an alternative segmentation requires definition of a similarity score between ground truth and a candidate border. Among the many scores are the overlap-based agreement ratio in [35] that uses the logical operation exclusive disjunction (symbolized XOR), the sensitivity and specificity, precision and recall, true positive rate, false positive rate, pixel misclassification probability [36], the Hausdorff and the Hammoude distances in [7], the weighted performance index [37], among others. A common fact with these scores is that they are computed from paired comparisons of borders. Recently an extension considering simultaneous comparison with multiple reference ground-truths (obtained by several dermatologists) was examined in [38].

In this paper, we will focus on three of the previous scores. In addition, an alternative score derived from the Hausdorff distance computation will be introduced. The first two scores considered are the sensitivity and specificity. Sensitivity and Specificity are statistical measures of the performance of a binary classification test, commonly used in medical studies. In the context of segmentation of skin lesions, sensitivity measures the proportion of actual lesion pixels which are correctly identified as such. Specificity measures the proportion of background skin pixels which are correctly identified. Given the following definitions:

TP: true positive, lesion pixels correctly classified as lesion,
FP: false positive, skin pixels incorrectly identified as lesion,
TN: true negative, skin pixels correctly identified as skin,
FN: false negative, lesion pixels incorrectly identified as skin,

and the number of pixels (#) in each of the above categories, the sensitivity and specificity are given by

(5) $$\begin{array}{c} \text{sensitivity}=\frac{\#\text{TP}}{\#\text{TP}+\#\text{FN}},\\ \text{specificity}=\frac{\#\text{TN}}{\#\text{TN}+\#\text{FP}}. \end{array}$$

The third score considered is the Hausdorff distance defined as follows. Let *ℳ* = {*m*_1_, *m*_2_,…, *m*_*z*_} and *𝒜* = {*a*_1_, *a*_2_,…, *a*_*n*_} denote the set of points belonging to the manually and automatically drawn contours *ℳ* and *𝒜*, respectively. The distance from *m*_*i*_ to its closest point in *𝒜* is given by

(6) $$\begin{array}{c} d\left(m_{i},𝒜\right)=\underset{j}{\min\;}||m_{\text{i}}-a_{j}||. \end{array}$$

The Hausdorff distance is the maximum of the distance to the closest points between the two curves,

(7) $$\begin{array}{c} d_{H}=\max\;\left\{\underset{i}{\max\;}\;d\left(m_{i},𝒜\right),\underset{j}{\max\;}\;d\left(a_{j},ℳ\right)\right\}. \end{array}$$

We convert *d*_*H*_ from pixels to millimeters for easier interpretation.

The forth and last score that will be considered is also based on the distance between the ground truth and candidate contour. Instead of taking the overall maximum value of the distance between contour points, as done in (7), we will compute the fraction of the contour pixels with an error lower than a predefined threshold *τ*

(8) $$\begin{array}{c} e\left(\tau \right)=\frac{1}{n+z}\left(\overset{z}{\underset{i=1}{\sum }}I\left(d\left(m_{i},𝒜\right)\leq \tau \right)+\overset{n}{\underset{j=1}{\sum }}I\left(d\left(a_{j},ℳ\right)\leq \tau \right)\right), \end{array}$$

where the indicator function *I*(·) = 1 when the condition is satisfied, 0 otherwise. 

The metric *e*(*τ*) is convenient in the sense that it allows the analyst to set an error tolerance during the comparison of a candidate border and the ground-truth reference. What is computed in (8) is a ratio of contour pixels matched below the error threshold tolerance. *e*(*τ*) is robust to the presence of local high disagreement between contours (outlier distances). For *τ* ≥ *d*_*H*_, *e*(*τ*) = 1, and we should therefore set *τ* < *d*_*H*_. Since the value of *d*_*H*_ is different for each lesion, and the disagreement between contours usually depends on how easy or difficult is the lesion to be segmented, a tradeoff value for *τ* should be set. In our experiments, we set *τ* = 0.5 mm. This applies for all lesions, and we belive it to be a reasonable tolerance for assessing the accuracy of the location of the contour of lesions seen at the high magnification provided by dermoscopes. In contrast to *d*_*H*_, a higher *e*(*τ*) score, indicates a better agreement of contours.

To conclude this section, it is worth noticing than when the sensitivity and specificity are computed, all pixels in the binary segmentation masks contribute to the final result. The Hausdorff distance, and the ratio of border samples with an error lower than the above threshold *τ* = 0.5 mm used in (8), are complementary scores to sensitivity and specificity, that are more oriented towards the quantification using the magnitude of a physical quantity as a measure of accuracy and tolerance of segmentation.

### 4.3. Algorithm Settings and Benchmark

During the classification, equal priors were assumed for skin and lesion. In order to speed up computation, all images were downsampled to 826 × 826 pixels using bilinear interpolation [39].

 For benchmarking, both unsupervised (automatic) and supervised segmentation methods are considered. 

#### 4.3.1. Fully Automatic Segmentation Methods

The first algorithm considered for benchmark was proposed in Celebi et al. (2008) [16]. The approach uses a region growing and merging technique called statistical region merging (SRM) [23] to segment the image. The SRM-based approach requires estimation of the color of the background skin. In our implementation, instead of placing four boxes at the corners of the image, as originality proposed, we used the location of the seed regions found by ICS to model the background skin. In practice, this avoids ambiguity, since the results would otherwise depend on how a square is cropped from the circular sector provided by dermoscopes, specially for lesions covering a large portion of the dermoscope.

The light-colored regions, that is, the regions whose mean color has a distance <60 to the background skin color are eliminated. The final result is obtained by removing the isolated regions and then merging the remaining regions, followed by a postprocessing stage [16]. SRM has an internal parameter *Q* that makes it possible to control the coarseness of the segmentation. Higher values of *Q* result in finer segmentation and thus the generation of more regions [23]. Specific details about the setting of the parameter *Q* are not reported by Celebi et al. in [16]. Using a trial and error procedure, a few candidate values for the parameter *Q* (as suggested in [23]) were applied to images from an independent training set. *Q* = 32 was found to be a reasonable choice. Clearly, a more in depth search using a more objective criterion for setting the optimal value of *Q* might lead to slightly different SRM results.

 The second automatic algorithm considered for benchmark purposes is the adaptive thresholding (AT) recently proposed by Silveira et al. (2009) in [7]. AT performs segmentation by comparing the color of each pixel with a threshold *τ*. A pixel is classified as lesion if it is darker than *τ*. First the algorithm search for the RGB channel which allows the best discrimination. It is assumed that the entropy provides the answer, and the channel with maximum entropy value is selected. In most dermoscopic images, the blue channel is selected. The histogram of the automatically selected color component is computed. For bimodal histograms, the threshold is automatically computed as the local minima between the maxima, plus a small offset to account for quantization issues. When the histogram has a single component, the threshold is obtained from the 5% percentile color of a squared region located at the center of the image, plus a constant offset. The default offset values presented in [7] are used in our implementation. 

#### 4.3.2. Supervised Segmentation Methods

As mentioned in the introductory section, supervised methods implies user interaction during the segmentation process. The way supervision can be used varies a lot. Supervision could be used in the initialization of the algorithm, or alternatively, at the end of the process, allowing for instance user interaction for manual correction of a proposed solution. Different algorithms are available in literature. For instance, in [7], the authors consider three edge-based methods that require only two mouse clicks for initialization. Very promising results are reported. In our study, however, we will prefer to deal with approaches more oriented to pixelwise classification along the lines of the proposed ICS method.

It is important to stress that in general a direct comparison between supervised and nonsupervised methods is unfair. Supervised methods are expected to outperform unsupervised methods, since they are given additional input information. But the reason why the proposed automatic ICS is compared with supervised methods in this paper has an important motivation: we would like to estimate how well a classification algorithm could perform if representative ground-truth (manually labeled) skin and lesion samples were used for training purposes.

In order to make the supervised analysis more trackable, a few practical experimental simplifications are done. First, we consider an estimated ground truth, given by the pixelwise majority voting of the independent segmentation masks provided by the three dermatologists (Section 4.1). In addition, the image is randomly sampled with a ratio corresponding to 1% of the total amount of pixels. Although low, this ratio provide a visually dense sampling of the image (*≈*22 pixels/mm^2^) that, given the high spatial resolution of the dermoscopic image, typically allows the selection of thousand of samples for both the background skin and the mole class.

Three supervised classifiers will be used for the segmentation (binary classification) task: the classic linear and quadratic discriminant analysis, LDA and QDA, respectively, and the support vector machines classifier (SVM) [40]. The SVM kernel used is the Gaussian radial basis function (RBF). The regularization term in the formulation of SVM and the kernel bandwidth are set using a traditional grid-search procedure with maximization of the 10-fold cross validation accuracy. Both LDA and QDA are also available in the proposed ICS algorithm. SVM is a nonparametric method based on a mathematical framework and presents several advantages compared to other pattern recognition methods, including the ability to handle large numbers or predictors with relatively small sample. Diagnosis of skin lesions is one among the many examples of use of SVM [41]. 

#### 4.3.3. Postprocessing

In order to minimize differences in the final border location due to differences introduced by specific postprocessing choices, mainly related to the criteria of exclusion of isolated regions and smoothing applied to draw the final border, the postprocessing discussed in Section 3 was applied to all the automatic and supervised segmentation methods, allowing a fair comparison.

### 4.4. Experiment  1: Evaluation of the Accuracy of the Automatic Selection of the Seed Regions

The location hypothesis that allows the initial sample selection for the proposed iterative segmentation algorithm plays a key role in the methodology. Intuitively, a good initialization increases the chance of a good final segmentation. In this first experiment, the interest is to experimentally verify if the proposed algorithm is able to accurately locate the skin and mole seed regions. For location accuracy assessment, here, the segmentation masks provided by the three dermatologists are combined to generate a reference map. For each image, we focus on the regions of agreement, where pixels are labeled as mole or skin by at least two of the three dermatologists. Ideally, we would like the algorithm to automatically place the initial small seed regions inside this “safe regions”. How it deviates from this ideal situation is then investigated in Figure 3. This figure shows the percentage of cases in the test set, where the seed regions are correctly located according with distinct accuracy levels. For assessment purposes, the lesions are grouped by diagnosis. For each lesion, the individual accuracies of both skin and mole seed regions are computed as the ratio between pixels correctly classified and the respective size of the seed. The “skin + mole” label in Figure 3 refers to the average value. Results in Figure 3 shows that for over 90% of the 122 dermoscopic lesions analyzed the seed regions are positioned in locations where at least two out of the three dermatologists would agree as accurate background skin or mole regions.

### 4.5. Experiment  2: Clinical Evaluation

When it comes to computing performance scores for assessment of the accuracy of the segmentation of skin lesions, like the scores presented in Section 4.2, the reference segmentation to be used as ground truth must be established.

According to previous studies, the use of the borders provided by a single dermatologist as ground truth should be avoided, since the solution for the same lesion by other dermatologists would exhibit a natural variability. A detailed discussion about this important point is beyond the scope of this work, but the reader is referred to [36, 42] for more details.

For the evaluation of the proposed method, the borders provided by three dermatologists are considered individually. An additional segmentation mask, generated by the pixelwise agreement in terms of the majority voting of the solutions provided by the three dermatologists is computed. The use of this additional segmentation is a simple attempt to obtain a more accurate estimation of the underline ground truth. Majority voting attributes the same weight for each dermatologist, which seems a reasonable hypothesis given their similar professional training. The segmentation mask generated by combination of the single solutions provided by dermatologists is expected to be more accurate than each of the individual solutions, because each manual solution is drawn independently, with an expected accuracy better than 50% (random guess). This is one of the key points usually exploited in the design of the so classed Ensemble methods in statistics and machine learning [43]. We believe majority voting would be a good attempt to estimate the underline ground truth, ideally considering manual borders from many independent dermatologists.

The average accuracy of segmentation is shown in Tables 1, 2, 3, and 4. The performance scores are computed using different references (marked “Ref.”) as ground truth: each of the manually drawn border by the three dermatologists, marked as “D1”, “D2”, and “D3”, plus an additional mask generated by the pixelwise majority voting of the three dermatologists's segmentations, marked as “All”. The accuracies are grouped in three blocks according to the nature of the segmentation solution: automatic methods and supervised methods. For reference purposes, scores corresponding to manual segmentation are included. For 2 out of the 100 benign lesions present in the test set, SRM did not provide a segmentation. These two lesions were excluded in this experiment.

Concerning the automatic methods, the main focus in this paper, Tables 1–4 shows that the proposed ICS method provide very competitive results when compared to SRM and AT, all the reference segmentations taken as ground truth, and for the different accuracy scores considered. In terms of sensitivity ICS scores above 91.8%, while SRM remains under 82.1%, with intermediate values for AT. On the other hand, the lower sensitivity scores of SRM are balanced by the higher specificities that lies above 98.0%, with ICS performing slightly lower at 96.7%. Surprisingly, the sensitivities and specificities of the automatic methods, in particular the proposed ICS, are very close to those of the dermatologists. However, the better performance of human segmentations when compared to the automatic methods is much clearer when the other two scores, the Hausdorff distance and the percentage of border pixels with an error lower of 0.5 mm, are considered. For these two scores, the ICS algorithm performs better than the SRM and AT alternatives.

Tables 1–4 clearly evidence the very good segmentation performance by the three supervised methods. Notice, however, that the supervised results were obtained after a dense sampling of training pixels, selected using the skin and lesion portions given by the majority voting solution (see details in Section 4.3.2). Thus, it is natural that in most of cases the performance of LDA, QDA, and SVM are even better than those of the single doctors. On the other hand, the results are interesting in the sense that they provide an idea where is the limit of the accuracy of a computer generated segmentation would be in the current test set, provided that individual training for each image based on the estimated underline ground truth was available and considering the current postprocessing employed. Somehow surprising is the poor performance of QDA compared to LDA. In any event, results show clear evidence that the nonlinear SVM classifier performs the best.

**Figure 4:**
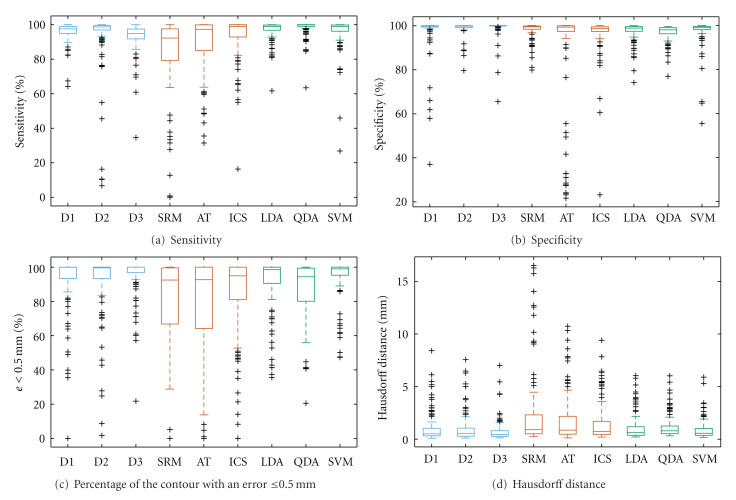
Dispersion of the accuracy of segmentation, quantified by the four distinct border measures, showing the performance of the proposed ICS method against the different segmentation methods analyzed. D1, D2, and D3 corresponds to the manual segmentations provided by dermatologists. SRM, AT, and ICS are automatic methods. LDA, QDA, and SVM are supervised methods that here include only for reference purposes. The accuracy scores are computed using the mask generated by majority voting of the manual segmentations provided by three dermatologists as ground-truth reference.

The above experimental results are based on the average values of the scores used for accuracy assessment. The dispersion of the scores is also an important factor to be analyzed. This is shown in Figure 4. Here, to reduce the amount of plots otherwise required for visualization of all data used to compute the averages in Tables 1–4, only the case corresponding to the use of the reference ground truth generated by majority voting is depicted (the scores corresponding to the row “all” in the previous tables). To understand the traditional box plots depicted in Figure 4, it is necessary to visualize its distinctive features. On each box, the central mark is the median, the edges of the box are the 25th and 75th percentiles, the whiskers extend to the most extreme datapoints the algorithm considers to be not outliers, and the outliers are plotted individually. Not surprisingly, the automatic segmentation methods show higher variability when compared to the borders manually drawn by dermatologists and the supervised methods tested. Overall, we once again appreciate the very competitive results provided by the proposed ICS algorithm, when compared to the alternative automatic methods considered, for all the four scores considered. Except for the Hausdorff distance, higher values indicate a more accurate segmentation. For the test set, ICS proved to be more stable as shown by the lower degree of dispersion of the scores, in the plots shown by the shorter distance between the lower and upper quartiles summarizing the data.

The contrast between the background skin and the lesion is expected to influence the accuracy of the delineation of the borders of the lesion. When evaluating the reasons why state-of-art CAD systems rejected analysing some lesions that would be of interest for dermatologists, Perrinaud et al. [3] observed that all instruments required the presence of “adequate contrast” between the lesion and surrounding nonlesional skin. Not surprisingly, borders manually drawn by different dermatologists tends to agree less when the delineation of the contours of the lesion is unclear. The same behavior is expected in automatic segmentation methods, impacting CAD systems.

**Figure 5:**
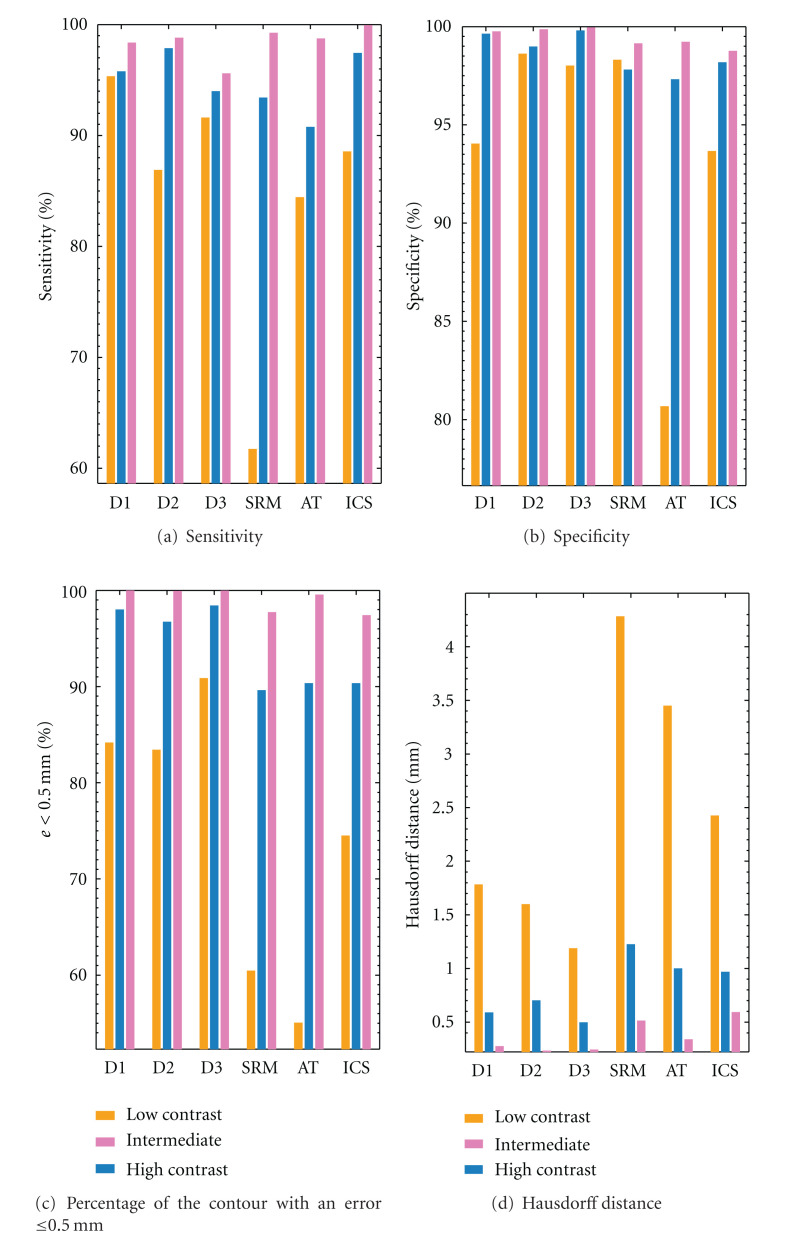
Average values of the accuracy of segmentation, quantified by the four distinct border measures, showing the performance of the proposed ICS method against the different segmentation methods analyzed. The lesions are grouped in three disjoint sets: low, intermediate, and high contrast between skin and lesion. The mask generate by majority voting of the manual segmentation by three dermatologists is used as ground-truth reference. The bars refer to low (orange), medium (blue), and high (pink) contrast lesions.

In order to get additional insights about the performance of the different segmentation methods for distinct levels of contrast, Figure 5 shows the average values of the accuracy of segmentations, quantified by the four distinct scores, focusing on the automatic segmentation methods analyzed. For reference purposes, the scores obtained from manual segmentation are included. In this complementary analysis, the set of 120 test lesions is grouped in three disjoint subsets (categories), here labeled as “low”, “intermediate”, and “high”, referring to increasing levels of contrast between background skin and lesion. In this study, the luminance component of the dermposcopic image is used to measure the contrast, quantified by the absolute value of the difference between the median of the lesion and skin pixels. For the purpose of computing the luminance difference between background skin and lesion pixels, the mask generated by majority voting of the manual segmentation of the three dermatologists was used.

The three subsets of lesions were automatically partitioned by minimizing the within-cluster sum of squares of the luminance values, according to the Euclidian distance criteria. The resulting subsets contained 49, 56, and 15 lesions, corresponding to the subsets labeled as low, intermediate, and high contrast, respectively. For indicative purposes, the respective values obtained for the contrast at the center of the clusters were 9.0, 21.9 40.4 (from 3 × 8 bits RGB images converted to *L*∗*u*∗*v* space). The high number of lesions with very low contrast stress the challenge of accurately segment the current test set, composed by many lesions of very low contrast (accounting for 41% of the 120 lesions), randomly selected, and previously unseen by the segmentation methods tested.

Figure 5 confirms that the accuracy of all the segmentation methods decreases when the contrast between lesion and skin is lower. In terms of segmentation scores, we observe that the proposed ICS presents very competitive results when compared to the SRM and AT algorithms, specially in presence of low contrast lesions. For instance, in terms of average sensitivity and specificity for this particular “low contrast” subset of lesions, ICS scores 88.6% and 93.7%, whereas AT scores 84.4% and 80.7%, when SRM scores 61.7% and 98.3%, respectively. Concerning these performance scores, we conclude that the best tradeoff is provided by ICS, followed by AT and SRM. The competitive performance of ICS is also confirmed when compared to the best performance by alternative algorithms, for both the Hausdorff distance (2.4 mm for ICS against 3.5 mm for AT) and the percentage of contour with an error ≤0.5 mm (74.5% for ICS against 60.5% for SRM). The good news is that when the level of contrast between skin and lesion increases, suggesting that the moles are easier to be segmented, the performance of the three methods increases considerably, remaining similar for all algorithms.

**Figure 6:**
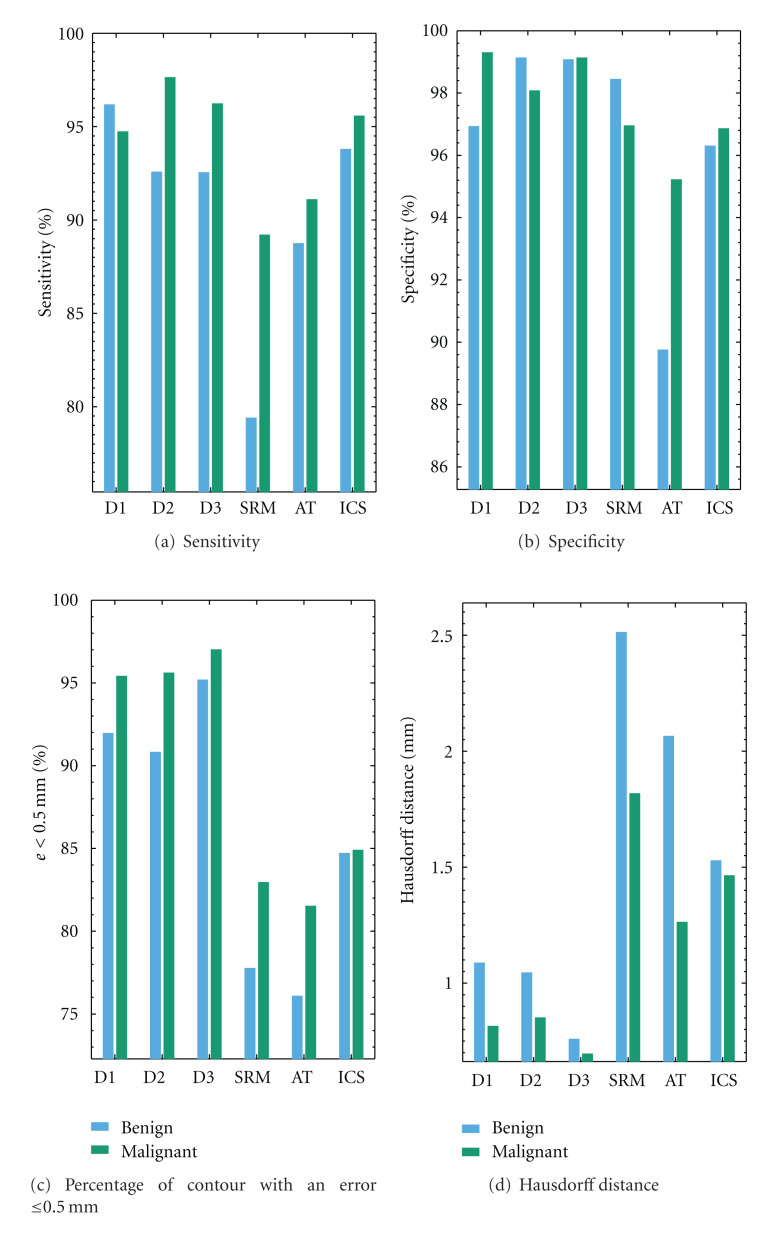
Average values of the accuracy of segmentations, quantified by the four distinct border measures, showing the performance of the proposed ICS method against the different segmentation methods analyzed. The lesions are grouped by histopathological diagnosis. The mask generate by majority voting of the manual segmentation of the three dermatologists is used as reference. The bars refer to benign (blue) and malignant (green) lesions.

We conclude this experiment examining the accuracy of the segmentation methods in terms of histopathological diagnosis. The lesions are grouped in two subsets according to benignity (98 lesions) or malignancy (22 lesions).  Figure 6 once again confirms the very competitive results provided by ICS. In the current test, set we observe that ICS performed similarly well for both benign and malignant lesions. This behavior of ICS was found consistent for the different accuracy scores. The differences in terms of performance for benign and malignant lesions is more remarkable for both the AT and SRM algorithms, that in general tended to score better on the malignant set, compared to their respective scores in the begin set.

### 4.6. Experiment  3: Convergence and Posteriors in ICS

The aim of the experiment is to provide additional details about the behavior of the proposed ICS framework during the classification. The two main aspects, (i) the number of iterations, and (ii) how the posteriors of the LDA and QDA classifiers are combined, are experimentally investigated.

**Figure 7:**
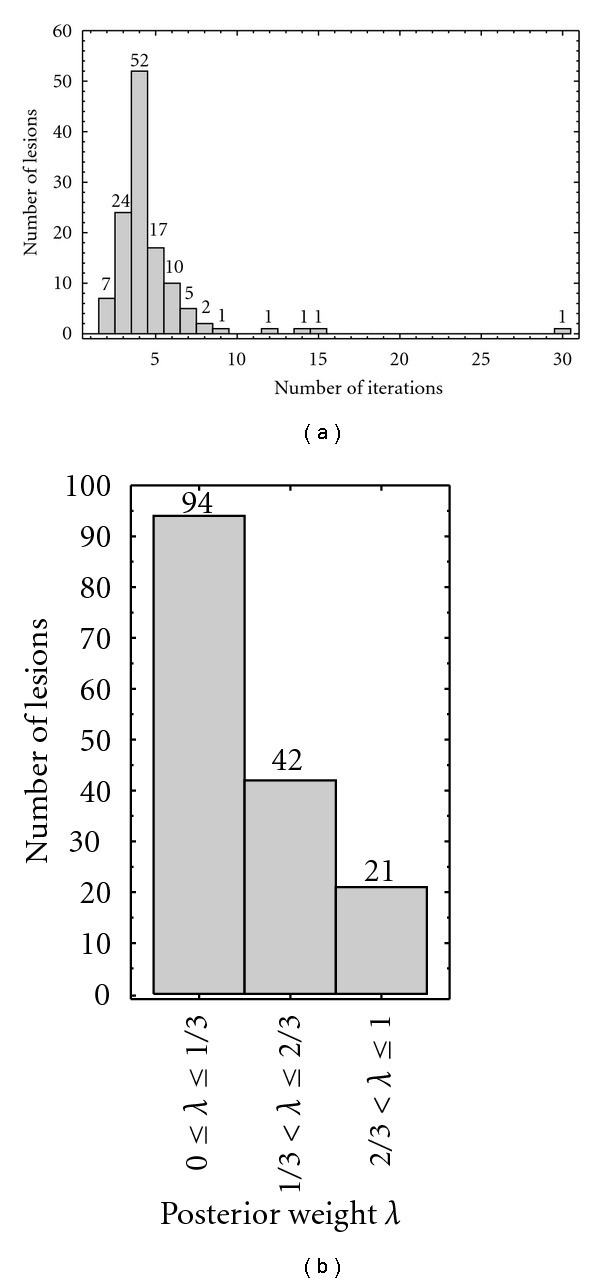
(a) Number of iterations for convergence and the (b) frequencies of the automatically selected value for the weight *λ* showing how the LDA (*λ* = 0) and QDA (*λ* = 1) posteriors where combined according to (1) in the proposed ICS algorithm. Results are summarized according to three intervals of possible *λ* values.

Figure 7(a) provides details about the number of iterations. On average, the method converged in 4.7 iterations. This suggests that just a few iterations are enough for modeling the statistics of the background skin and lesion. During experiments, we set the maximum number of iterations *n* = 30. If not converged at iteration *n* = 30, the iterative classification was stopped, and the last result used. As shown in Figure 7(a), this occurred only for one lesion, suggesting that the proposed method is very likely to converge for the task of segmentation of dermoscopic images.

How the posteriors are combined in the proposed ICS framework is analyzed in Figure 7(b). For summarization purposes, we group the values of *λ*_*k*_ in (1) in three intervals (i) 0 ≤ *λ*_*k*_ ≤ 1/3 (ii) 1/3 < *λ*_*k*_ ≤ 2/3, and (iii) 2/3 < *λ*_*k*_ ≤ 1 and count the number of lesions that had, for some of the iterations *k* = {1,…, *n*}, a *λ*_*k*_ value inside the intervals. As shown in Figure 7(b), for most of the cases (94 lesions) the proposed ICS framework selected low values of *λ*_*k*_, suggesting the preference for LDA. This fact is not surprising in the current implementation. Since the separability between the initial samples from the skin and lesion seeds is usually good, the accuracy of the seed regions is very high in most cases (*≈*100%), and in case of identical accuracy for both LDA and QDA, *λ*_*k*_ in (1) is taken as the lowest value possible, thus intentionally privileging the simpler LDA (for the reasons discussed in Section 2.4). It is also worth noticing that when LDA was used in the context of supervised segmentation, LDA tended to perform slightly better than QDA (Tables 1–4). But selection of low values for *λ*_*k*_ is not always the case, in particular, for other 42 and 21 lesions in the two remaining intervals shown in Figure 7(b), indicating intermediate preferences between LDA and QDA, and a stronger preference for QDA, respectively. In the current analysis, the total number of lesions contained in the three intervals is bigger than the number of lesions in the test set. This is due the fact that *λ*_*k*_ is optimized for each iteration, so in this analysis, a given lesion may account in more than one of the intervals considered until reaching convergence.

**Figure 8:**
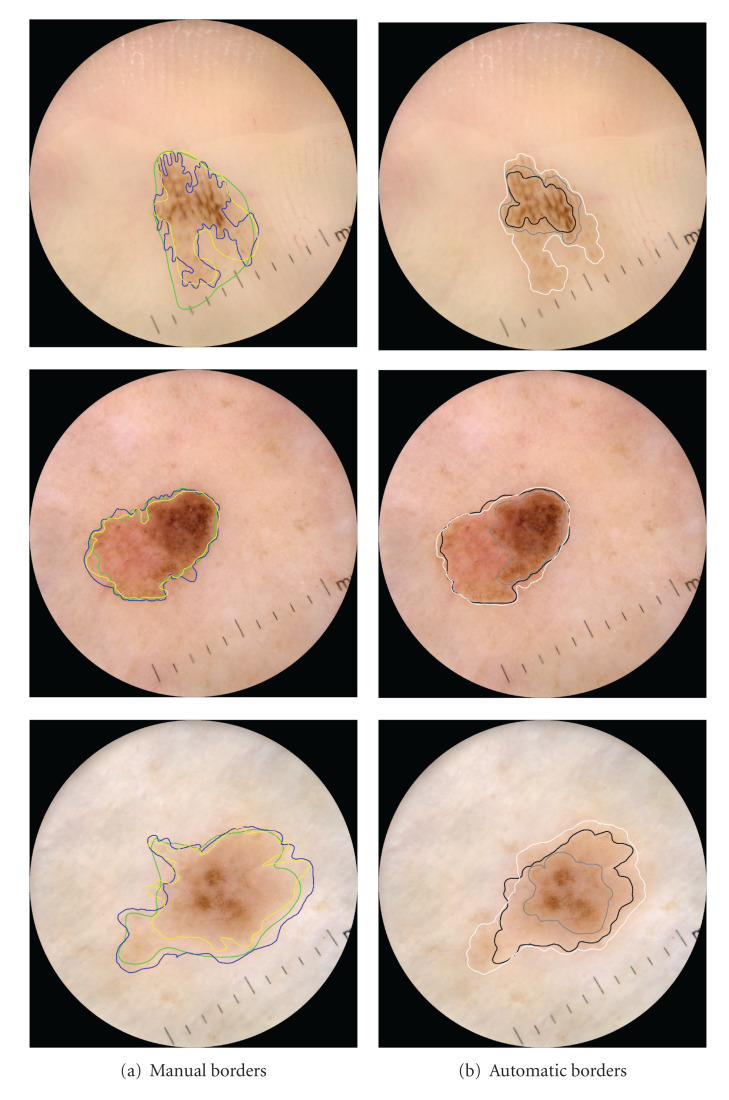
Example of lesions in the test set and the border provided by (a) the three dermatologists and (b) the automatic borders by AT (black border), SRM (gray border), and the proposed ICS algorithm (white border). None of the methods performed better in all cases.

**Figure 9:**
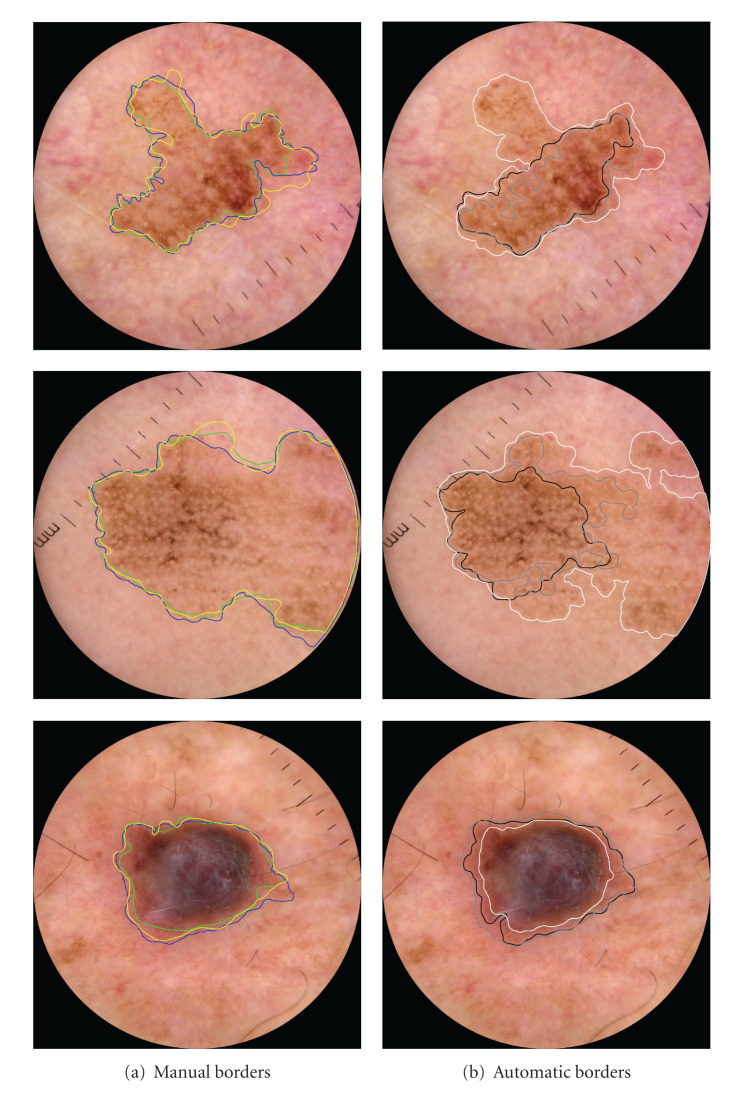
Example of lesions in the test set and the border provided by (a) the three dermatologists and (b) the automatic borders by AT (black border), SRM (gray border), and the proposed ICS algorithm (white border). None of the methods performed better in all cases.

We conclude our analysis showing in Figures 8 and 9 a few examples of pigmented skin lesions from the test set, and the manual border drawn by the dermatologists, the proposed ICS, and the alternative AT and SRM methods. Note that none of the methods performs better in all cases.

## 5. Conclusions and Final Remarks

We have presented an automatic algorithm for segmentation of pigmented skin lesions. The technique is primarily developed for analysis of images acquired by a portable dermatoscope attached to a consumer-grade digital camera.

In contrast to other automatic segmentation techniques, the proposed ICS algorithms relies on specific assumptions about the image acquisition, in particular the approximate location and color of the skin and lesion. The assumptions are simple in nature and are designed for the specific problem of segmentation of dermoscopic images. The main purpose is a safe selection of initial small seed regions corresponding to skin an lesion portions that through iterative classification leads to the final segmentation mask.

The clinical accuracy assessment using 122 dermoscopic images, randomly selected, with ground-truth lesion borders manually drawn by three dermatologists suggests competitive segmentation results when the proposed ICS algorithm is compared to alternative automatic segmentation methods. The improvements are particularly remarkable for lesions with low contrast between background skin and lesion. In addition, in the current test set the proposed algorithm was found to perform similarly well for both begin and malignant lesions.

Overall, the proposed framework is simple and flexible enough to allow testing with different classifiers. Compared to a traditional 1-D histogram-based segmentation, the proposed approach uses all the RGB color information available. In addition, since the proposed segmentation framework is essentially classification based, it could eventually accommodate additional input features such as contextual information. Usually, convergence was reached in a few iterations. The algorithm is relatively fast (takes about 1 min.), and the processing time depends essentially on the choice of the classifiers, whose posteriors are combined automatically.

We believe that the suggested framework is general enough to be useful for analysis of other kind of images acquired by different equipments, adapting the initial assumptions about the geometry of acquisition and color of the lesion of interest to the specific problem at hand. In addition, despite not directly envisaged in this paper, it appears also that the proposed method could be easily modified to accommodate user iteration, for instance, by manual placement of seed regions, rendering the proposed method even more robust. 

We conclude reminding that it is important to keep in mind that the effect of border detection error upon the accuracy of a computer aided diagnosis system can only be validated when used as a part of a final diagnostic system.

## Figures and Tables

**Table 1:** Average accuracy of segmentation, quantified by the sensitivity (%), showing the performance of the proposed ICS method against alternative solutions. Borders manually drawn by three dermatologists, and their agreement, are considered as the ground-truth reference for accuracy assessment.

	Manual by doctors			Fully automatic			Supervised		
Ref.	D1	D2	D3	SRM	AT	ICS	LDA	QDA	SVM
D1	—	86.9	85.4	79.2	87.3	91.8	93.4	95.3	92.2
D2	89.5	—	87.1	79.6	87.1	93.4	94.3	96.8	93.6
D3	92.7	91.0	—	82.1	90.0	94.0	96.1	97.7	95.1

All	95.9	93.5	93.2	81.2	89.2	94.1	97.0	98.5	96.0

**Table 2:** Average accuracy of segmentation, quantified by the specificity (%), showing the performance of the proposed ICS method against alternative solutions. Borders manually drawn by three dermatologists, and their agreement, are considered as the ground-truth reference for accuracy assessment.

	Manual by doctors			Fully automatic			Supervised		
Ref.	D1	D2	D3	SRM	AT	ICS	LDA	QDA	SVM
D1	—	98.3	98.4	98.0	91.6	96.7	97.6	96.9	97.7
D2	96.1	—	97.6	98.3	90.3	96.3	96.9	96.2	96.8
D3	96.4	97.9	—	98.0	90.7	96.2	96.8	96.2	96.8

All	97.4	98.9	99.1	98.2	90.8	96.4	97.3	96.7	97.4

**Table 3:** Average accuracy of segmentation, quantified by the percentage of contour with an error ≤0.5 mm, showing the performance of the proposed ICS method against alternative solutions. Borders manually drawn by three dermatologists, and their agreement, are considered as the ground-truth reference for accuracy assessment.

	Manual by doctors			Fully automatic			Supervised		
Ref.	D1	D2	D3	SRM	AT	ICS	LDA	QDA	SVM
D1	—	84.5	87.1	75.7	75.0	81.1	86.3	81.8	88.8
D2	84.5	—	86.8	76.8	74.8	84.5	88.2	84.8	90.1
D3	87.1	86.8	—	78.5	77.1	82.7	88.5	83.3	90.6

All	92.6	91.7	95.5	78.7	77.1	84.8	91.5	87.5	94.4

**Table 4:** Average accuracy of segmentation, quantified by the Hausdorff distance (mm), showing the performance of the proposed ICS method against alternative solutions. Borders manually drawn by three dermatologists, and their agreement, are considered as the ground-truth reference for accuracy assessment.

	Manual by doctors			Fully automatic			Supervised		
Ref.	D1	D2	D3	SRM	AT	ICS	LDA	QDA	SVM
D1	—	1.50	1.35	2.50	1.91	1.68	1.40	1.47	1.17
D2	1.50	—	1.31	2.42	2.14	1.56	1.22	1.33	1.14
D3	1.35	1.31	—	2.43	1.93	1.61	1.25	1.37	1.13

All	1.04	1.01	0.75	2.39	1.92	1.52	1.09	1.20	0.86
